# Clinically amyopathic dermatomyositis associated with cutaneous ulcerations: a case-based review

**DOI:** 10.1097/MS9.0000000000001669

**Published:** 2024-01-03

**Authors:** Alice Viana De Jesus, Jean Marcos De Souza

**Affiliations:** Faculdade de Ciencias Medicas, Universidade Estadual de Campinas (UNICAMP), Campinas, Brazil

**Keywords:** case report, dermatomyositis, myositis, skin ulcer, therapeutics

## Abstract

**Introduction and importance:**

Dermatomyositis (DM) is an autoimmune myopathy primarily affecting both muscles and skin. When muscle weakness is not clinically apparent, but characteristic skin lesions are present, the condition is referred to as clinically amyopathic dermatomyositis (CADM).

**Case presentation:**

The authors present the case of a 52-year-old female with a typical DM rash, interstitial pneumonia, and multiple skin ulcers. The skin biopsy was consistent with DM, and there were no signs of muscle involvement. Myositis-related and myositis-specific autoantibodies were also negative. Significant improvement was not observed until the patient received successive monthly pulses of methylprednisolone and the introduction of methotrexate. This treatment regimen allowed for the complete tapering of prednisone and resulted in sustained disease control.

**Clinical discussion:**

In addition to the case presentation, a narrative literature review was conducted using the MEDLINE database, and an evidence-based treatment flowchart is proposed. CADM is a subtype of DM, related to higher incidences of interstitial lung disease, skin vasculopathy and malignancy. When ulcers or interstitial pneumonia are present, treatment should be early and aggressive. Active screening for neoplasms is recommended, particularly within the first 5 years.

**Conclusion:**

The authors presented a case of seronegative CADM featuring skin vasculopathy, successfully treated with consecutive methylprednisolone pulses. Our literature review emphasized the importance of focused CADM management trials, highlighting the need for further research.

## Introduction and importance

Highlights

- Clinically amyopathic dermatomyositis (CADM) is a form of DM without weakness.
- CADM with skin ulcers is most often associated with anti-MDA5 autoantibodies.
- Our patient presented with ulcers, but tested seronegative.
- Recurring methylprednisolone pulses facilitated rapid reduction of oral prednisone.
- Intensive treatment and malignancy screening are recommended for those patients.

Dermatomyositis (DM) pertains to the large family of inflammatory myopathies (IM) that mainly affect muscle and skin. Its prevalence is 1–6 per 100 000 adults, with a preference towards women^1^, and the age at diagnosis in adults ranges from 40 to 60 years, on average^1^.

Muscle involvement findings include symmetrical muscle weakness of the pelvic and scapular girdles, elevation of muscle enzymes and suggestive findings on muscle biopsy and electroneuromyography^2^.

The most typical cutaneous manifestations, among others, include erythema of the eyelids (heliotrope sign), face, and upper trunk, as well as violaceous scaly lesions on the extensor joint face (Gottron’s lesions) and scaly erythema with periungual thickening on the fingers’ nails^3^. Other symptoms that may be present include pruritus and calcinosis. The lesions can cause hyperpigmentation or cutaneous hypopigmentation, leading to poikiloderma. Palmar papules and ulcers over the joints are also described^4^.

Clinically amyopathic dermatomyositis (CADM) accounts for about 20% of adult cases^3^ and 5–20% of juvenile DM^4^. It is defined as a subgroup of patients with classic dermatomyositis skin manifestations, in the absence of symptoms or signs of myositis on physical examination for at least 6 months after rash onset^3,5^.

Specific autoantibodies are found in about 50–70% of DM cases, and the antibody most associated with cutaneous ulcerations is anti-MDA5^6^. Skin ulcers are usually regarded as indicative of a worse prognosis^7^.

In this case-based review, we describe the case of a patient with seronegative CADM and prominent cutaneous ulcerations, a form of disease usually related to anti-MDA5 antibodies. We also highlight the role of consecutive methylprednisolone pulses, allowing fast oral glucocorticoid tapering. For the surveillance of disease activity, International Myositis Assessment and Clinical Studies Group (IMACS) core set measures were used, including MYOACT, MITAX and the IMACS response criteria^8,9^.

Next, we present a literature review on CADM, highlighting the role of aggressive immunosuppression in severe forms and the search for occult neoplasia. This study fulfills the guidelines from CARE^10^ and SCARE^11^ for case reporting.

For the review, we searched the MEDLINE database for reviews published in English between 2000 and 2023. The utilized terms were: “dermatomyositis” and “skin ulcers”. For the management section, we searched the terms “dermatomyositis” and “therapeutics” for review articles published in the last 5 years and filtered by “best match” and “most recent” tools in the PubMed interface.

## Case presentation

We report the case of a 52-year-old female, otherwise healthy. For the 9 months preceding the visit, she complained of polyarthralgia and erythematous rash on her hands, fingers, chest, and face. In her hands, she also reported painful ulcers. Additionally, she lost 7 Kg and was experiencing dysphagia, despite denying limb muscle weakness.

**Figure 1:**
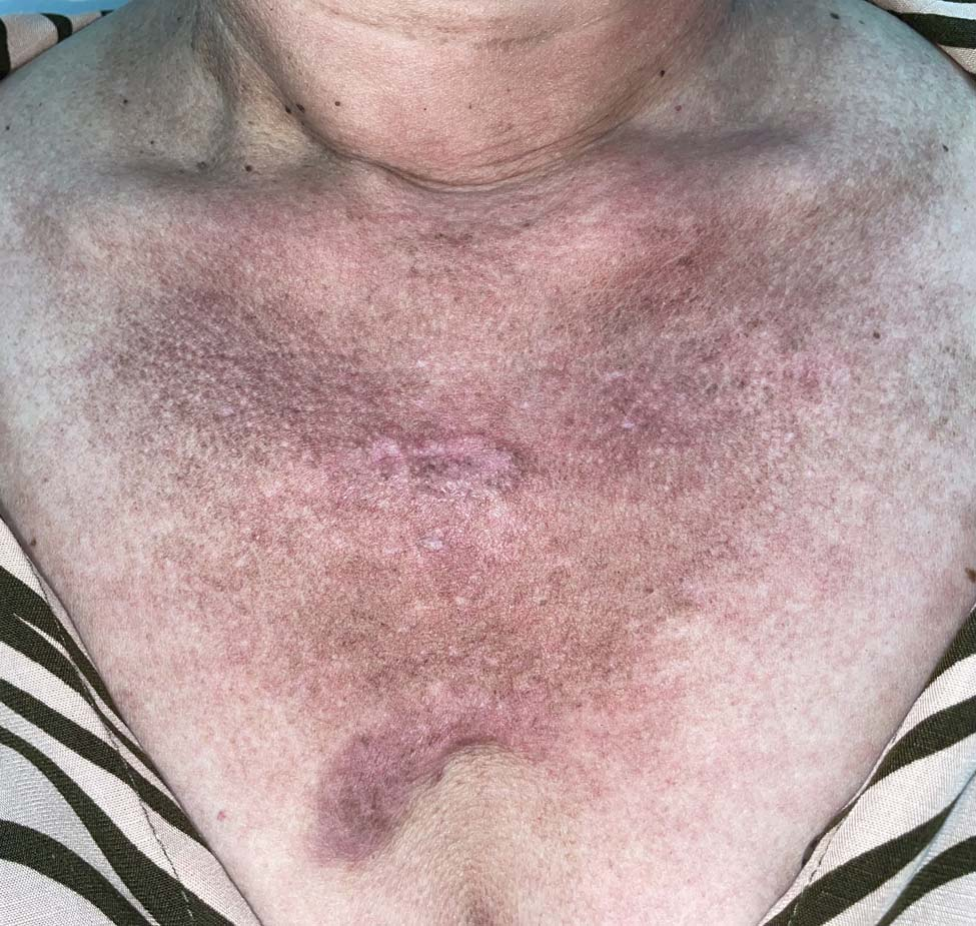
Photograph of the patient before the intravenous treatment: An erythematous and hyperchromic rash (V-sign) can be seen, indicating active disease.

**Figure 2:**
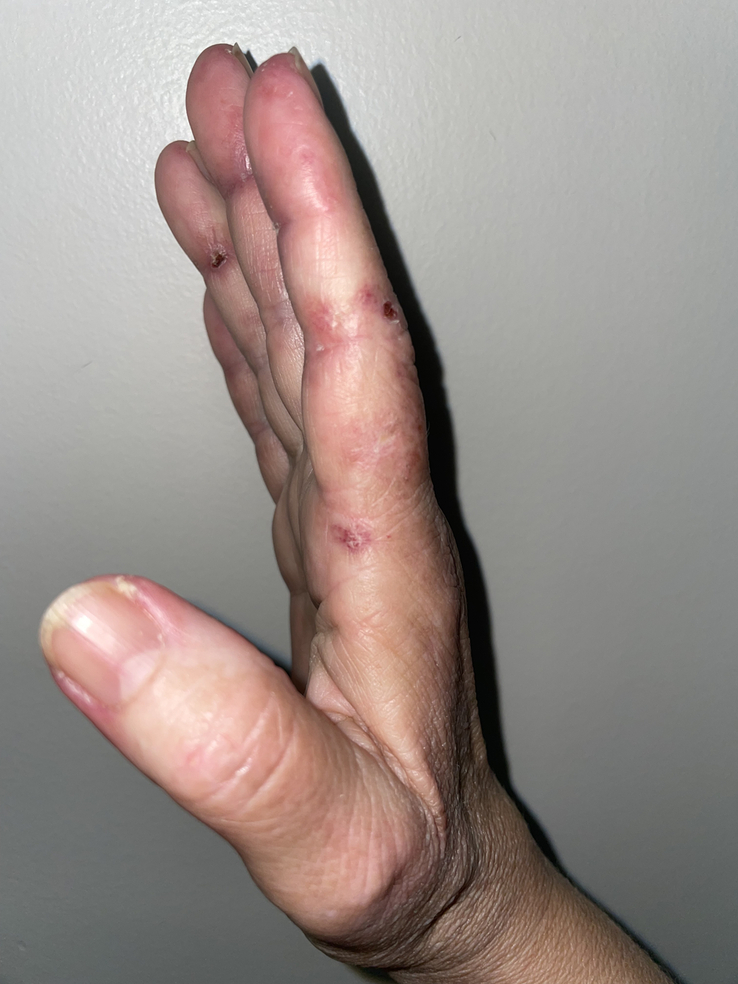
Photograph of the patient’s hand before the intravenous treatment: Small ulcers in the finger’s and nailfold erythema can be seen, indicating vasculopathic disease activity.

The physical examination during the first visit depicted an underweight and apparently ill patient. There were erythematous and lightly scaly plaques covering the hands, torso, and eyelids, suggesting Gottron’s papules, heliotrope rash, shawl’s sign, and V-sign (Fig. 1). The hands and feet exhibited Reynaud’s phenomenon, with various painful ulcers (Fig. 2). The reported joints were tender and swollen, and muscle strength was preserved in all limbs.

Laboratory workup revealed elevated inflammatory markers, normal creatine phosphokinase, and no other organic failure. Antinuclear antibody (ANA) was positive with a nuclear dense fine speckled pattern, titre 1:320. Regarding antibodies, Anti-Ro52, Anti-PM/Scl, Anti-Ku, Anti-SAE, Anti-TIF1γ, Anti-NXP2, Anti-MDA5 and all Anti-synthetase were negative. Antibodies for systemic lupus erythematosus and rheumatoid arthritis were also negative. Finally, thorax computed tomography showed nonspecific interstitial pneumonia, and skin biopsy showed interface dermatitis with hyperparakeratosis and mucin deposits, suggesting CADM. Of note, the patient fulfilled the most recent classification criteria of IM^12^ as definite IM, subgroup amyopathic DM.

Initially, she was treated with prednisone (up to 40 mg, ~0.7 mg/kg/day) and hydroxychloroquine for ~3 months, with no response. At this moment, MITAX score was 24/54 and MYOACT was 12/60, with prominent skin manifestations. The possibility of cancer-associated myositis was considered, but our screening was negative. The decision was made to introduce pulse therapy with methylprednisolone at a daily dose of 1 g for 3 days. Oral prednisone was tapered to 10 mg/day, and methotrexate at a weekly dose of 10 mg was initiated. One month later, skin lesions showed only slightly improvement, and the patient complained of dry cough and no weight gain. IMACS total improvement score (TIS) summed to 17.5, not even reaching minimal improvement. Therefore, a decision was made to undergo new pulse therapy with methylprednisolone (3 g total). The methotrexate dose was also increased to 15 mg per week, and the oral glucocorticoid was reduced to 5 mg per day. One month later, TIS showed moderate improvement, MITAX was 4/54 and MYOACT 2/60. Because slight erythema of the chest remained 4 months later, in order to completely taper the prednisone, methotrexate was raised to the maximum dose (20 mg/week). The patient was seen recently, 1 year from the beginning of the treatment, without signs of disease activity.

## Clinical discussion

The literature search on skin ulcers in DM yielded 15 articles, out of which eight were unrelated to myositis or not in English. The remaining seven articles were used for the literature review. For the management section, we employed the “best match” and “most recent” filters on PubMed, resulting in 193 search results. We selected the first 20 articles as the basis for our review and consulted additional articles within this repository as needed.

Here, we present a clinical case of CADM with prominent cutaneous lesions and ulcerations and interstitial lung disease, negative for anti-MDA5, the main antibody related to this clinical picture^13^. We also highlight the good clinical response to the early combination of sequential pulse methylprednisolone therapy and methotrexate, allowing fast tapering of oral glucocorticoids with sustained disease control.

The pathogenesis of DM is not yet fully understood. Several genetic factors have been described, such as specific HLA alleles and cytokine polymorphisms^3,14^. An infectious trigger may also be present, such as group B Streptococcus, Toxoplasma spp., Coxsackie virus, Enterovirus, and Parvovirus^15^. Exposure to ultraviolet radiation, smoking, and medication are also debated^1^.

Regarding the pathogenesis, antibodies directed against endothelial cells deposit at the perimysial zone^14^. This leads to the formation of a membrane attack complex in the vessels, stimulating the release of cytokines. Consequently, there is an influx of inflammatory cells into the perimysial muscle, including B cells, CD4+ T cells, and plasmacytoid dendritic cells. Thus, there is necrosis and ischaemia of muscle fibres, resulting in a reduction in endomysial capillary density^2^. It is worth noting that perifascicular atrophy is characteristic of DM, even in the absence of local inflammatory infiltrate^16^. When ulcers are present, the same mechanism applies but is directed toward the vessels in the dermis^17^. In summary, vascular injury, mediated mainly by humoral mechanisms, appears to play a crucial role in the pathogenesis of DM^18^.

The classical findings of the disease include Gottron’s sign and heliotrope rash^1,2^. Other lesions are also compatible, such as erythematous plaques, nailfold changes and poikiloderma^1^. Periungual telangiectasias, dystrophic cuticles, superficial erosions, and cutaneous calcinosis may also be present^2^. Palmar papules and ulcers over the joints are more commonly found in individuals with anti-MDA5-associated disease^3^. They have been associated with higher resistance to immunosuppressive therapy, indicating a poor prognosis^17^.

Overt myopathy is not a feature in CADM, and because dysphagia is attributed mainly to oropharyngeal weakness, this feature, although present in our patient, is infrequent^19^. Interstitial lung disease occurs in up to 50% of patients with DM. Although very severe forms of ILD have been described in seronegative patients^20^, the involvement occurs at an even higher frequency in the subgroup associated with the MDA5 antibody, reaching 90–95%, and 50–80% progress to a rapidly progressive form (RP-ILD). This complication has a high mortality, reaching up to 50%^21^, and is generally refractory to immunosuppression^5^.

The diagnosis is made based on clinical and laboratory findings^14^. The skin biopsy depicts vacuolar interface dermatitis and mucin deposition in the dermis^2^, along with perifascicular myofibrillar atrophy and necrosis^14^. Other histological findings that may be present include hyperkeratosis, epidermal atrophy, perivascular infiltrate rich in CD4+ T lymphocytes, and endothelial damage with capillary loss^1^.

The incidence of cancer in DM patients is higher than in the general population^22^. Among the neoplasms associated with dermatomyositis, notable ones include ovarian, breast, colon, melanoma, nasopharyngeal, lung, lymphomas, leukaemias, bone tissue, and joint tissue cancers^2,22^. Neoplasms can be diagnosed before, concurrently, or up to 5 years of follow-up, prompting regular screening^23^. Patients with CADM are also prone to malignancy, highlighting the need for surveillance^3^.

Among the autoantibodies found in the immunological spectrum of CADM, the following can be mentioned: anti-SAE, anti-Mi2, anti-TIF1, anti-NXP2, and anti-MDA5. The latter is associated with more severe cutaneous vasculopathy^6^.

**Figure 3:**
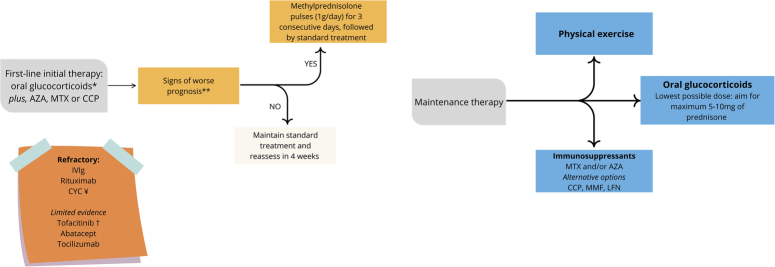
Suggested management algorithm for clinically amyopathic dermatomyositis. AZA, azathioprine; CCP, cyclosporin; CYC, cyclophosphamide (monthly intravenous pulses); IVIg, intravenous human immunoglobulin; LFN, leflunomide; MMF, mycophenolate mofetil; MTX, methotrexate. * 0.5–1.0 mg/kg/day for 4–8 weeks and taper. ** Interstitial lung disease, severe dysphagia, or cutaneous ulcerations. Consider in patients with elevated ferritin or lactate dehydrogenase^21^. Consider monthly pulses until, at least, moderate response is achieved. ¥ Best evidence for interstitial lung disease. † Best evidence for skin disease and interstitial lung disease.

Regarding management (Fig. 3), the use of oral glucocorticoids is considered first-line treatment for initial therapy, at a dose of 0.5–1.0 mg/kg/day for at least 4 weeks^24^. Despite their efficacy, corticosteroid therapy does not achieve a complete response in over 50% of patients^24^. Pulse therapy with methylprednisolone in DM is particularly indicated in severe cases, such as cutaneous ulcerations, severe dysphagia, interstitial lung disease, or prominent myopathy, at a dose of 1 g per day for 3 consecutive days^14,24^.

Although the role of sequential methylprednisolone pulses has not yet been established in myositis in general, this strategy has been used successfully in a retrospective study with immune-mediated necrotizing myopathy^25^. In our patient, the approach seemed favourable, allowing fast tapering of the oral prednisone.

Among immunosuppressants, azathioprine and methotrexate are the most commonly used in clinical practice^14^, while cyclosporine, tacrolimus, leflunomide, mycophenolate, cyclophosphamide, and tofacitinib represent therapeutic options^24^. The combination of corticosteroids with immunosuppressors is preferred to mitigate the long-term adverse effects of corticosteroid therapy^5^.

In refractory cases or when immunosuppressive drugs are contraindicated, such as during infections, the use of intravenous immunoglobulin should be considered^24^. In the context of clinical refractoriness, rituximab is usually the preferred agent^26^.

Physical exercise and/or physiotherapy are recommended throughout all phases of the disease and have been shown to be beneficial and safe, with the potential for improving cardiorespiratory function and muscle strength^27,28^.

We summarized the suggested treatment approach in Figure 3.

In summary, our patient presents mainly ulcerative and vasculopathic CADM, usually related to anti-MDA5, but with negative autoantibodies. Clinical improvement and fast prednisone tapering were observed after sequential methylprednisolone pulse therapy. While the seronegativity of our case is unusual, it is not the first reported^13^, limiting the novelty factor. The short follow-up is also an issue, as cancer-associated myositis might emerge. We tried to attenuate our limitations by providing a detailed literature review and a treatment strategy, highlighting the possible role of sequential methylprednisolone pulse therapy.

## Conclusion

While anti-MDA5 CADM patients are prone to skin vasculopathy and RP-ILD, even seronegative cases can develop these severe issues. Despite limited evidence, our case responded well to sequential methylprednisolone pulses. More research and randomized trials focused on this population are crucial.

### Patient perspective

In the last visit, the patient was asked to provide a brief description of her experience. She said that the scariest factor was not understanding what was going on. The pain and dysphagia also were important factors for stress. When the team offered intravenous therapy, she felt as she was undergoing chemotherapy and that was also freighting. Now, after a year of treatment, she said she feels almost as she did before, but the pain and fatigue abate from time to time. She recognizes the burden of living with a chronic condition, known for its ups and downs.

## Ethical approval

The study was approved by the Ethics Committee of the Universidade Estadual de Campinas (UNICAMP), Campinas (Sao Paulo), Brazil. Number of protocol: 73830923.6.0000.5404, number of the approval statement: 6.340.812.

## Consent

Written informed consent was obtained from the patient for publication of this case report and accompanying images. A copy of the written consent is available for review by the Editor-in-Chief of this journal on request.

## Sources of funding

None.

## Author contribution

A.V.J. contributed to the conceptualization, planning of the methodology, literature investigation, patient record analysis, writing and revision of the draft, and approval of the final version of the manuscript. J.M.S. contributed to the conceptualization, planning of the methodology, writing and revision of the draft, approval of the final version of the manuscript, and overall supervision.

## Conflicts of interest disclosure

All authors declare no conflicts of interest.

## Research registration unique identifying number (UIN)

None.

## Guarantor

Jean Marcos de Souza.

## Data availability statement

Data sharing is not applicable to this article.

## Provenance and peer review

Not commissioned, externally peer-reviewed.
